# The missing mechanistic link: Improving behavioral treatment efficacy for pediatric chronic pain

**DOI:** 10.3389/fpain.2022.1022699

**Published:** 2022-10-14

**Authors:** Maya L. Jotwani, Ziyan Wu, Claire E. Lunde, Christine B. Sieberg

**Affiliations:** ^1^ Department of Psychiatry and Behavioral Sciences, Biobehavioral Pain Innovations Lab, Boston Children's Hospital, Boston, MA, United States; ^2^ Pain and Affective Neuroscience Center, Department of Anesthesiology, Critical Care, Pain Medicine, Boston Children's Hospital, Boston, MA, United States; ^3^ Department of Psychiatry, Harvard Medical School, Boston, MA, United States; ^4^ Nuffield Department of Women's and Reproductive Health, Medical Sciences Division, University of Oxford, Oxford, United Kingdom

**Keywords:** pediatric chronic pain, behavioral intervention, pain treatment, mechanistic clinical trial (MCT), neuroimaging, fNIRS (functional near infrared spectroscopy), multimodal MRI

## Abstract

Pediatric chronic pain is a significant global issue, with biopsychosocial factors contributing to the complexity of the condition. Studies have explored behavioral treatments for pediatric chronic pain, but these treatments have mixed efficacy for improving functional and psychological outcomes. Furthermore, the literature lacks an understanding of the biobehavioral mechanisms contributing to pediatric chronic pain treatment response. In this mini review, we focus on how neuroimaging has been used to identify biobehavioral mechanisms of different conditions and how this modality can be used in mechanistic clinical trials to identify markers of treatment response for pediatric chronic pain. We propose that mechanistic clinical trials, utilizing neuroimaging, are warranted to investigate how to optimize the efficacy of behavioral treatments for pediatric chronic pain patients across pain types and ages.

## Introduction

Pediatric chronic pain is a widespread, global burden, with epidemiological data estimating that up to 83% of children experience chronic pain, depending on the pain type (1). Pediatric chronic pain is particularly complex to manage and treat due to developmental changes in the nervous system (2). Specifically, changes in the function and density of nociceptive receptors and myelination, along with alterations in endogenous inhibitory control, contribute to changing pain response and modulation (2). The consequences of pediatric chronic pain are significant, as children with chronic pain report experiencing a worse quality of life and more missed school days (3) Chronic pain conditions also impose significant stress on parents and overall family functioning (4–6).

The transition from acute to chronic pain is thought to be due to sensitization of the central nervous system (CNS) leading to pain amplification (7). Ascending and descending pain modulatory systems constitute distributed brain regions including the somatosensory cortices, prefrontal and anterior cortices, amygdala, nucleus accumbens, thalamus, and brainstem (8–12). Neuroimaging studies have consistently reported the structural and functional associations of these brain regions with pain perception (13–15).

In addition to neurological factors, the pain experience is modified by psychological and social factors (e.g., pain catastrophizing, parent protective responses), which contribute to pain-related disability and pain treatment outcomes (16–21). However, there is a general lack of understanding of how these factors interact with other biobehavioral mechanisms (e.g., brain metrics, inflammatory biomarkers) to impact pain treatment outcomes. A recent commission on pediatric pain by the Lancet Child / Adolescent Health Commission (2) proposed four goals to improve the lives of children and adolescents with pain as well as their families including: (i) to “make pain matter”; (ii) “make pain understood”; (iii) “make pain visible”; and (iv) “make pain better”. To achieve these goals, there is a need to better elucidate the mechanisms contributing to behavioral treatment response.

Currently, the literature has indicated mixed efficacy for behavioral treatments for pediatric pain (studies include children aged 7 to 18 years old) (22–27). There is a clear need to move beyond randomized controlled trials (RCTs) of behavioral pain treatments that solely rely on patient self-reported outcomes. Instead, conducting mechanistic clinical trials using mixed methods (e.g., neuroimaging, self-report, biomarkers) will allow for a more comprehensive assessment of treatment responders. We posit that personalized pediatric pain treatment has remained elusive in large part due to a dearth of research in this area, specifically mechanistic clinical trials. Of note, there are only 23 published studies on PubMed that describe their study design as a “mechanistic clinical trial”, with only three focused specifically on adult pain conditions and none focused on pediatric pain (28–30).

In this mini review, we focus specifically on how neuroimaging has been used to identify the biobehavioral mechanisms contributing to chronic pain and how this modality could be instructive in mechanistic clinical trials as a marker of treatment response.

## Treating pediatric chronic pain

Psychological interventions, including cognitive behavioral therapy (CBT), mindfulness-based therapy, and acceptance and commitment therapy (ACT), have demonstrated efficacy for treating pediatric and adult chronic pain (17, 21–27, 31–45). Many studies found significant reductions in pain intensity, functional disability, anxiety, and depression post-treatment (see the recent Cochrane review (26) and others (22, 25, 27, 32, 36, 40, 42)).

Psychological therapies for pediatric populations have mixed efficacy for pain-related outcomes. Such behavioral treatments have moderate effects on reducing pain intensity post-treatment with Standardized Mean Difference (SMD) effect sizes ranging from −0.43 to −0.57 (22, 25) and a Needed to Treat ratio of 2.32 – meaning that two people needed to be treated for one to benefit from the therapy compared to controls (27). Therapies have been found to have a small to moderate beneficial effect on disability post-treatment (SMD −0.45 to −0.34) (22, 25) and a limited to no effect on depression (SMD −0.05 to −0.07) and anxiety (SMD −0.16 to −0.15) outcomes (22, 25). However, these improvements (except for disability, SMD −0.27) were not maintained in long-term follow-up (22, 25). It remains unclear why psychological therapies may have short-term effects but may not be maintained in the long-term. Mechanistic clinical trials could help to address this gap.

Defined by the NIH, “*a mechanistic clinical trial is designed to understand a biological or behavioral process, the pathophysiology of a disease, or the mechanism of action of an intervention*” (46). We posit that utilizing neuroimaging in mechanistic clinical trials to examine the interaction of brain and behavior is critical. This approach will enable us to better understand the processes and pathophysiology of pediatric chronic pain, as well as the mechanisms of action of behavioral pain treatment.

## The potential for neuroimaging: investigating pediatric chronic pain mechanisms and treatment

Over the past few decades, several studies (47–59) have examined the relationship between chronic pain and neurocognition *via* performance-based neuropsychological assessment. Results have been mixed. Most of the studies show that patients with chronic pain or neuropathy-related conditions such as fibromyalgia, back pain, and diabetes have neurocognitive impairments including relatively poor processing, psychomotor speed, attention and executive function, memory, and learning (47–54). Still, other studies have failed to find an association between chronic pain and certain neurocognitive processes (55–59).

Furthermore, many observational studies have investigated the role of the CNS in influencing behavior and brain functions and demonstrated the significant impact of its ascending and descending inhibitory systems on pain modulation and perception (60–63). In addition, advances in modern neuroimaging techniques enable objective assessment of brain structural and functional properties, allowing for the identification of brain-based markers of chronic pain (64–83). These markers could be important therapeutic targets.

Chronic pain is a complex phenomenon, and its underlying neural mechanisms are not fully understood. Current pain assessment methods primarily rely on observations of individuals' symptoms and context of their pain (84–86). As such, it has been a significant clinical challenge to accurately assess pain from the subjective evaluations of young patients (87) or patients who are cognitively impaired or developmentally delayed (88). The development of advanced neuroimaging techniques (e.g., MRI, fNIRS) has the potential to supplement subjective clinical pain evaluation to improve accurate diagnoses and treatment plans for patients with chronic pain. Additionally, unlike measures such as self-report, neuroimaging could elucidate specific neurological mechanisms underlying different pain conditions and pain treatment response. For example, Dr. Maria Fitzgerald and her team have demonstrated the utility of a range of neuroimaging techniques to investigate neonatal pain processing. Specifically, her team have used MRI, EEG, and fNIRS to find that when exposed to innocuous and noxious stimuli, newborns demonstrate distinct patterns of functional brain activations (89–93). These results have elucidated meaningful insights about appropriate clinical measurement and treatment of infant pain.

In addition, the complex and diverse presentation of pain conditions leads to difficulty for directing patients to specific treatments for their pain condition. As a result, there are a number of emerging neuroimaging studies aimed at better delineating pain classifications and underlying biological mechanisms to develop more personalized treatment (83, 94–96). This approach has been used for years in other fields – many psychiatry neuroimaging studies are underway or have been conducted to identify brain-based markers and predictors of treatment response for anxiety and depression. These researchers highlight the goal of using these neurological markers and predictors to develop more personalized pharmacological therapies and to select suitable candidates for different treatment approaches (97–103). A similar approach using neuroimaging to identify treatment response markers is needed to improve efficacy of pain treatments for pediatric pain patients.

### Using multimodal magnetic resonance imaging (MRI) for adult chronic pain

The majority of the research using neuroimaging for chronic pain has focused on adults (refer to Supplementary Table 1). Structural magnetic resonance imaging (MRI) and diffusion tensor imaging (DTI) have been used to investigate brain structure and tissue architecture differences associated with chronic pain. Specifically, one voxel-based morphometry study showed gray matter (GM) volume and density reductions in multiple cortical areas, including the cingulate cortex and insular cortex, as well as subcortical regions, such as the thalamus, in adult patients with chronic pain relative to healthy controls (69). The role of white matter fiber tracts has also been investigated in relation to pain, with numerous DTI studies demonstrating microstructure abnormalities measured by fractional anisotropy, mean diffusivity, axial diffusivity, and radial diffusivity in patients with chronic pain (104–110).

Extensive task-based and resting-state functional magnetic resonance imaging (fMRI) studies have investigated a range of chronic pain conditions including neuropathic pain, fibromyalgia, chronic low back pain, headache, migraine, and chronic osteoarthritis (70–81) (refer to Supplementary Table 1). After different non-pharmacological pain treatments, including acupuncture, psychological therapies, and cranial electrical stimulation, many studies show changes in brain markers. Specifically, activation and functional connectivity changes have been found in the somatosensory and motor cortices (71–73), anterior cingulate cortex (74, 75), insula (76–78), posterior cingulate cortex (79), prefrontal cortex (80), orbitofrontal cortex (81), and thalamus (83).

### Using functional near-infrared spectroscopy (fNIRS) for adult chronic pain

More recently, pain studies have used functional near-infrared spectroscopy (fNIRS) (111–118), a non-invasive optical technology that quantifies cortical concentration changes in oxygenated and deoxygenated hemoglobin from the absorption of near-infrared light through cortical tissues (90). Unlike fMRI, fNIRS allows the investigation of brain hemodynamics in a clinical setting, making it suitable for broader use, such as during surgery (119–123).

For example, Gentile and colleagues utilize fNIRS to investigate brain responses in adults with fibromyalgia across several studies (114, 115, 124). They have consistently found that fibromyalgia patients have significantly lower task-evoked brain activation and electrical activity in the motor cortex compared to healthy controls (114, 115, 124). These consistent findings not only reveal potential motor and pain-related circuit dysfunction in fibromyalgia but also validate the reproducibility of fNIRS investigations in pain.

### Using multimodal MRI and fNIRS in pediatric chronic pain

#### Structural and functional MRI: pain processing

Structural and functional brain properties involved in pain-evoked behavioral responses have been extensively examined in adults, however, only a limited number of neuroimaging studies have been conducted in pediatric patients (117, 118, 121, 125–140) (refer to Supplementary Table 1). A few studies have used fMRI to show that adults and newborn infants have similar neural activity during pain processing. Goksan et al. (2015) found that activated brain regions during noxious stimulation were very similar for both infants and adults – with activations in the cerebellum, insula, putamen, and anterior cingulate cortex, to name a few (131). In addition to this overlapping activity, there were also distinct differences such as hypoactivation of the amygdala and orbitofrontal cortex in infants relative to adults (131). Furthermore, Goksan and colleagues went on to investigate the role of the descending pain modulation system (DPMS) (130). Infants with greater functional connectivity in the DPMS prior to stimulation had lower noxious-evoked brain activity (130). These results were not replicated for the Control Network nor the Default Mode Network – highlighting a specific mechanism of the DPMS during pain experiences (130).

Many studies have investigated GM volume and resting-state functional connectivity (FC) in children (ages 10 to 18) with complex regional pain syndrome (CRPS) (125–127, 132, 134, 140) (refer to Supplementary Table 1). Results show that relative to healthy controls, pediatric patients with CRPS have altered structural and functional properties in various networks related to cognitive and affective functioning (125–127). In addition, CRPS patients showed reduced GM volume and increased resting-state FC in the subcortical basal ganglia of the sensorimotor network (125, 126). Decreased resting-state FC of the habenula, a brain structure linked to pain processing, has been found in children and adolescents (aged 10–17) (134). One study found decreased task-evoked brain activity in many regions such as the precentral gyrus, inferior frontal gyrus, supramarginal gyrus, and postcentral gyrus for CRPS patients (aged 8–20) when completing a fearful face paradigm (132). Widespread cortical changes were also observed for CRPS patients (aged 9–18) directly after noxious stimulation, with increased activation in areas involved in sensation and emotional processing, as well as decreased activation in frontal and parietal lobes, and in limbic system structures (140). In recovery from evoked pain, CRPS patients had persistent decreased activation in frontal, parietal, temporal cortices and the hippocampus (140).

Such brain abnormalities have also been found in children with migraine (aged 9 to 17), with demonstrated GM volumetric abnormalities in the frontal and temporal lobe, fusiform gyrus and putamen compared to healthy volunteers (128). A study recently investigated structural and functional properties in adolescents (aged 10 to 24) with peripheral nerve injury of the ankle (133). In the ankle injury cohort, there was reduced GM in the bilateral somatosensory cortices compared to healthy controls and decreased resting-state FC in the nucleus accumbens, amygdala, and the periaqueductal gray – regions associated with affect and pain modulation (133). In addition, the ankle injury cohort showed changes in white matter integrity, with the superior parietal lobule, inferior parietal lobule, and anterior thalamic radiation showing significant changes in mean diffusivity compared to healthy controls. These tracts are associated with pain processing and sensory integration (133). For peripheral neuropathic pain, one study found that children and adolescents aged 11 to 18 had stronger resting-state FC between the right amygdala and right dorsolateral prefrontal cortex, and enhanced resting-state FC between the right amygdala and left angular gyrus compared to controls. These trends were correlated with lower pain intensity, reflecting pain inhibition-related resting-state FC differences (136).

#### Treatment effects on brain imaging measures

Several studies have investigated the effect of treatment on brain functional connectivity and gray matter structures for pediatric pain patients (125, 127). Simons et al. (2014) found that pediatric CRPS patients (aged 10 to 17) who underwent an intensive psychophysical treatment program had significant decreases in connectivity between the left amygdala and motor cortex, parietal lobe, bilateral cingulate and one lobule of the cerebellum (127). In turn, these connectivity changes were correlated with decreases in pain-related fear after treatment, suggesting the potential for amygdala connectivity as an indicator of psychological treatment response. Another study also showed treatment-induced connectivity changes for pediatric CRPS patients (125). Pre-treatment, CRPS patients had negative connectivity between the dorsal-lateral prefrontal cortex and periaqueductal gray, whilst post-treatment, a positive connectivity was observed (125). The authors propose this change may indicate that successful treatment response is related to increased synchronicity between the structures (125). Further, the patients had greater cortical thickness in the dorsal-lateral prefrontal cortex and greater subcortical GM volumes in several subcortical structures after treatment (125).

#### fNIRS: pain processing and treatment

A small, but growing number of studies have used fNIRS imaging to investigate pain processing and pain treatment response for children and infants, with a focus on acute and procedural distress. Pettersson et al. (2019) measured pain perception in healthy newborn infants (mean age of 39.9 weeks) during a hip examination, a routine medical examination that is thought to cause discomfort for infants (135). Compared to heart auscultation examinations, which are non-painful, the hip examination evoked greater oxygenated hemoglobin on bilateral somatosensory cortices. This oxygenation was concurrent with greater Premature Infant Pain Profile-Revised scores, which assesses procedural pain in infants (135). Recently, Yuan et al. (2022) assessed nociceptive prefrontal functional activation undergoing circumcision in neonates (aged 1–2 days) (139). They found that prefrontal activation significantly increased during noxious events (e.g., local injection) and decreased with non-noxious events (e.g., before incision) (139). Karunakaran et al. (2022) investigated the effects of continuous remifentanil on cortical hemodynamics in pediatric patients (mean age of 15.8 years) in response to catheter ablation (121). They reported that the placebo-controlled group showed greater oxygenated brain activations in inferior and superior medial frontopolar cortices compared to the remifentanil group (121).

Furthermore, the effects of pain-alleviating strategies for newborn infant procedural pain has been investigated using fNIRS in a few studies (118, 137, 138, 141). Ren et al. (2022) found that a white noise intervention did not significantly change cerebral oxygen saturation of newborn infants (aged 37–42 weeks) during a blood sampling procedure (137). Two studies investigated the analgesic effects of glucose or sweet solution administration to newborn infants during painful procedures (118, 138). Bembich et al. (2015) found that glucose did not evoke significant cerebral oxygenation changes compared to a breast-feeding group during a heel prick procedure (aged 38–41 weeks) (118). Intriguingly, Beken et al. (2014) found that glucose significantly increased cerebral blood volume after a blood sampling procedure for newborn infants (median age of 38 weeks) (138). Bembich et al. (2015) also found that breast-feeding evoked less intense pain behaviors and caused greater generalized cortical activation in newborn infants (118). Lastly, one study found that skin-to-skin contact reduces the oxygenated hemoglobin activation of infants (aged 30 weeks) during a blood sampling procedure compared to no skin-to-skin contact (141). It is possible that these pediatric acute pain fNIRS studies could inform further research for utilizing fNIRS for pediatric chronic pain treatment.

Improved understanding of the specific neural mechanisms underlying pediatric chronic pain and treatment response will allow for more targeted psychological and pharmacological pain treatments. For example, specific behavioral interventions may improve functional connectivity in key brain regions implicated in chronic pain symptomatology, such as between the amygdala and prefrontal structures (142). This insight could direct patients with specific neural markers related to amygdala connectivity to this intervention. In this way, neural treatment response predictors and markers have the potential to be transformative for pediatric chronic pain treatment.

## Where to next?

Unanswered, yet important questions remain, including: Do psychological interventions have similar neurological effects on adults and children with chronic pain? Which pain condition and at which age is a certain behavioral intervention the most efficacious? We suggest the next step for answering these questions is to deeply phenotype the psychological treatment effects through neuroimaging. By starting with neuroimaging, we can elucidate the neurological markers that may predict treatment response. These neurological markers, in conjunction with patient self-report, can be used to formulate pain treatment approaches. Considering the multiple, diverse mechanisms of pain, mechanistic clinical trials using multiple methods including neuroimaging is warranted for comprehensive understanding of treatment response (143).

### Mechanistic clinical trials

Given the complex pathophysiological mechanisms of pain, different approaches have been used to examine pain's biological and behavioral etiology. Mechanism-based approaches, which target patients' specific pain-related characteristics, could allow for the development of a more personalized treatment approach (143). Pain researchers and drug regulators highlight that conventional clinical trials are insufficient for effective clinical analgesic development, as these trials do not account for heterogeneous pain mechanisms (143). Furthermore, the evidence base for pediatric chronic pain mechanisms needs to be expanded to support the conceptualization of pharmacologic and nonpharmacologic pain treatment trials. Findings from adult chronic pain studies cannot be relied on as the mechanisms of pediatric pain may be significantly different with the interplay of a developing nervous system (144). The Lancet Child / Adolescent Health Commission's 2020 report on pediatric pain highlights the need for alternate approaches beyond the RCT to address gaps in evidence-based treatment and to guide clinical practice (2). Notably, there is a dearth of mechanistic clinical trials for behavioral pain treatment and none for pediatrics. By studying the mechanisms behind treatments, the mechanistic clinical trial approach could also help identify risk factors of chronic pain to aid clinical efforts to prevent the onset of chronic pain (2).

Early mechanistic clinical trials have discussed shortcomings of this approach, including the failure to identify apparent differences in treatment response, the lengthy process, and general participant discomfort for pain assessment. However, recent mechanism-based studies have simplified methods and used more patient-friendly paradigms for assessing pain mechanisms (145). For example, Wang et al. implemented two double-blind, placebo-controlled trials that investigated the mechanisms of serotonin–norepinephrine reuptake inhibitor (SNRI) antidepressant medication for adults with persistent depressive disorder using MRI (145). Using this mechanism-based approach, they found that antidepressants decreased functional connectivity compared with placebo within a thalamo-cortico-periaqueductal network, which has previously been associated with the experience of pain (145). This reduced functional connectivity was also correlated to improvements in depressive symptoms and pain symptom severity in the medication group. Wang and colleagues have revealed the utility of using a mechanism-based approach. By using MRI, Wang's group were able to investigate neurological mechanisms underlying symptom severity changes during antidepressant treatment. This approach can help develop more specific targets for antidepressant therapeutics and bolster our understanding of depression in general.

Mechanistic clinical trials are gaining momentum (29, 30, 97, 98, 146–157). One feasibility study has been successfully conducted, which assessed the feasibility of examining the beneficial metabolic effects of bariatric surgery in adults using a mechanistic clinical trial design (151). Five ongoing mechanistic clinical trials with published protocols aim to improve treatment outcomes in adult patients. These include optimizing pain interventions for patients with fibromyalgia (30), identifying biomarkers for pharmacoresistant depression treatment response (97), diet as a treatment for acute coronary syndrome (148), mental stress on coronary heart disease (147), and ventilator-induced diaphragm dysfunction (146). We suggest a similar model should be used for pediatric chronic pain to better understand the neural mechanisms underlying pain processing, higher intervention accuracy, and neurological and biomarker advancement.

The saturation of RCTs and significant gaps in knowledge in the adult and pediatric chronic pain literature warrants a new research approach. Mechanistic clinical trials that incorporate neuroimaging offer an avenue to investigate brain-behavior interactions underlying pediatric pain treatment. Understanding the underlying processes will allow for developing personalized treatments with more optimal outcomes.

## Discussion

Despite the significant prevalence and impact of pediatric chronic pain, there remains a lack of understanding of etiology and effective treatment. The psychological components of pain and pain-related functional outcomes are well-documented and have precipitated a rise in studies investigating psychological treatment interventions for pediatric chronic pain. Thus far, results are promising, with treatments correlated to decreased pain interference and intensity, improved affective measures, reduction in disability, and improved quality of life. However, these effects are far from consistent, and it remains unknown why some patients experience improvements while others do not.

Neuroimaging, particularly fNIRS imaging, is a promising technique to elucidate the underlying neurological mechanisms of pediatric chronic pain. Although fNIRS has its inherent limitations including relatively lower spatial resolution and lack of sufficient penetration depth of near infrared light for capturing subcortical brain activities (158), the technology offers unique and significant benefits. Advantages include the portability of the device and low sensitivity to head motion for monitoring pain evaluation in clinical settings (159–162). In addition, development of innovative techniques, such as machine learning, which detects patterns, rules, and causal dependencies in large chronic pain study data sets, have enabled objective data evaluations of chronic pain by incorporating temporal and spatial features of fNIRS imaging data (163, 164). Integration of machine learning in pain research makes it possible to investigate neural predictors of treatment responses, and even provide recommendations for appropriate, effective treatments for chronic pain.

Future research should utilize neuroimaging techniques and integrative analysis to investigate the neurological mechanisms behind pediatric chronic pain and treatment response. In addition, large, multicenter mechanistic clinical trials investigating the neurological and psychosocial mechanisms of change for psychological treatment interventions is warranted. Only once the heterogeneous mechanisms of pediatric chronic pain and treatment response are understood can we begin to develop precision medicine to optimize care for all pediatric chronic pain patients.
