# Supplementary material for: The missing mechanistic link: Improving behavioral treatment efficacy for pediatric chronic pain
Source: Front Pain Res (Lausanne). 2022 Oct 14;3:1022699. doi: 10.3389/fpain.2022.1022699 (PMC9614027; doi:10.3389/fpain.2022.1022699)
Supplement: Supplementary file 1 [file Table1.docx]

Table 1: Multimodal MRI and fNIRS findings in pain populations.

| **References** | **Type of Pain** | **Study Population** | **Neuroimaging Modalities** | **Treatment/**  **Intervention** | **Major Findings of Brain Measures** |
| --- | --- | --- | --- | --- | --- |
| Schmidt-Wilcke et al, 2008 [68] | Migraine | 35 patients with migraine (mean age= 32.4 years); 31 healthy controls (mean=32.3 years) | Structural MRI | N/A | Migraine patients vs. controls: decreased GM volume in anterior and posterior part of the cingulate cortex, and right insular cortex. |
| DaSilva et al, 2007 [104] | Migraine with and without aura | 12 migraine patients with aura (mean=33.8 years); 12 migraine patients without aura (mean=36.0 years); 12  healthy controls (mean=31.0 years) | DTI | N/A | Migraine patients vs. controls: lower FA in migraineurs in the thalamocortical and ventral trigeminothalamic tracts.    Migraine patients without aura vs. controls: lower FA in the ventrolateral periaqueductal GM. |
| Gomez-Beldarrain et al, 2015 [105] | Episodic; chronic migraine | 19 episodic migraine patients (mean=41.37 years); 18 chronic migraine patients (mean=43.78 years); 15 healthy controls (mean=45.73 years) | DTI | N/A | Chronic migraine patients vs. controls at 6 months follow-up assessment: significantly lower FA values in the right anterior insula, bilateral cingulate gyri, and right uncinate fasciculus |
| Qin et al, 2019 [106] | Migraine without aura | 50 migraine patients without aura (mean=38.7 years); 50 healthy controls (mean=39.5 years) | DTI | N/A | Migraine patients without aura vs. controls: decreased FA in the vermis VI extending to the bilateral lobules V and VI of the cerebellum; higher AD, MD, and RD in the right inferior cerebellum peduncle tract; reduced GM volume and increased AD, MD and RD in the spinal trigeminal nucleus. |
| Szabó et al, 2012 [107] | Migraine | 21 female migraine patients (mean=34.65 years);  17 healthy female controls (mean=33.27 years) | DTI | N/A | Migraine patients vs. controls: reduced FA, increased MD and increased RD in the right frontal white matter cluster. |
| Yuan et al, 2012 [108] | Migraine without aura | 21 migraine patients without aura (mean=32.4 years);  21 healthy controls (mean=31.6 years) | DTI | N/A | Migraine patients without aura vs. controls: reduced FA values in the genu and the splenium of CC; decreased inter-hemispheric resting state FC of ACC; reduced FA values of the genu of CC correlated with the decreased inter-hemispheric rsFC of the ACC. |
| Teepker et al, 2012 [109] | Cluster headache (CH) | 7 male patients with episodic CH (mean=43.14 years);  7 healthy controls (mean=50.43 years) | DTI | N/A | Patients with CH vs. controls: increased FA in bilaterally white matter of brainstem, frontal lobe, temporal lobe, occipital lobe, internal capsule, and on the right side of thalamus and cerebellum. |
| Hayes et al, 2017 [110] | Trigeminal neuralgia (TN) | 37 patients with TN (23-70 years); 28 healthy controls (23-67 years) | DTI | N/A | TN patients vs. controls: decreased FA, increased MD and RD in middle and posterior cingulum. |
| Kim et al, 2013 [70] | Fibromyalgia | 21 female patients with fibromyalgia (mean=51.3 years); 11 healthy controls (mean=46.5 years) | Task-based functional MRI | N/A | Patient group showed increased brain activations in bilateral supramarginal gyrus, contralateral insula, IFG, thalamus, calcarine, ipsilateral cerebellum and STG. |
| Timmers et al, 2019 [71] | Chronic low back pain | 14 patients with chronic low back pain (mean=42.4 years); 14 healthy controls (mean=41.7 years) | Resting-state and task-based functional MRI | Cognitive-behavioral based therapy | Pre-treatment: patient group showed increased activation in right posterior insula and increased deactivation in mPFC. Pre- vs. post-treatment: patient group showed a decrease in right postcentral/ supramarginal gyrus and precentral gyrus, and an increase in activity in the precuneus.    Pre-treatment vs. 6 months follow-up: patient group showed decreased activations in right angular/inferior parietal lobe, right postcentral, right middle frontal/dorsolateral PFC, right inferior frontal/ventrolateral PFC as well as left middle frontal gyrus.    Post-treatment vs. 6 months follow-up: patient group showed decreased activation in right posterior cingulate cortex.    In controls: no effects of time in the posterior insula and mPFC cluster. |
| Gustin et al, 2010 [72] | Complex  regional pain syndrome (CRPS) | 20 patients (29-65 years): 15 patients with CRPS type I, 5 patients with  CRPS type II | Task-based functional MRI | Therapy (NMDA receptor antagonist + morphine) vs. morphine + placebo | Pre vs post-treatment: Therapy group had reduced activation in contralateral primary somatosensory cortex and ACC. Pain relief was associated with decreased activation of the contralateral primary SSC and secondary SSC.    Therapy group vs. morphine + placebo: Therapy group had reduced activation in contralateral primary SSC. |
| Sanders et al, 2015 [73] | Osteoarthritis (OA) of the carpometacarpal (CMC) joint | 19 patients (50-80 years) with painful OA of the CMC joint of the right hand | Task-based functional MRI | Naproxen vs placebo | Treatment vs. placebo: Decreased brain activities in bilateral primary SSC, thalamus, and amygdala. |
| Koeppe et al, 2004 [74] | Fibromyalgia | 6 female patients (mean=56 years) with fibromyalgia | Task-based functional MRI | 10 mg of the  5-HT3 receptor antagonist tropisetron | Pre- vs. post-treatment: Reduced activation in contralateral primary SSC, contralateral posterior insula, and ACC. |
| Petzke et al, 2013 [75] | Fibromyalgia | 90 females (18–55 years) with fibromyalgia | Task-based functional MRI | 13-weeks milnacipran treatment (200 mg/day) vs. placebo | Pre- vs. post-treatment: Increased pain-evoked brain activity in the caudate nucleus, anterior insula and amygdala.    Treatment vs. placebo: greater pain-evoked activity in the precuneus/PCC. |
| Harte et al, 2016 [76] | Fibromyalgia | 42 patients with fibromyalgia (18-75 years); 20 healthy controls | Task-based functional MRI | Pregabalin treatment vs. placebo | Patients with fibromyalgia vs. controls: Greater activation in the right anterior insular cortex.    Pre- vs. post-treatment: Decreased activation in bilateral anterior insula. |
| Jensen et al, 2012 [77] | Fibromyalgia | 43 female patients (43-48 years)  with fibromyalgia syndrome | Task-based functional MRI | Cognitive Behavioral Therapy | Pre- vs. post-therapy: Increased activations in the ventrolateral prefrontal/lateral orbitofrontal cortex. |
| Taylor et al, 2013 [78] | Fibromyalgia | 46 patients with fibromyalgia (mean=50.8 years) | Task-based functional MRI | Cranial electrical stimulation therapy | Active device vs. sham device: Decreased activation in posterior cingulate gyrus, cingulate gyrus, ACC, and thalamus. |
| Smallwood et al, 2016 [79] | Comorbid chronic low back pain | 25 patients with chronic low back pain (32-55 years) | Resting-state and task-based functional MRI | Chronic pain-focused ACT; health education control (HEC) | ACT treatment vs. HEC control: Decreased activation in middle frontal gyrus, inferior parietal lobule, insula, ACC, PCC, and superior temporal gyrus. |
| Grazzi et al, 2010 [80] | Chronic migraine with symptomatic medication overuse | 13 female migraine patients; 11 female healthy controls (30-44 years) | Task-based functional MRI | fMRI scans were performed  before and after withdrawal of acute medication | Before withdrawal: the right supramarginal gyrus, the right inferior and superior parietal cortex were hypoactive.  After withdrawal: Activity recovered to almost normal at 6 months after withdrawal of the offending medications |
| Li et al, 2017 [81] | Migraine without aura | 100 patients with migraine (17-45 years); 46 healthy controls | Resting-state functional MRI | Verum acupuncture | Patients with migraine vs. controls: Increased activations in posterior insula and putamen/caudate, and reduced activations in rostral RVM/TCC    Pre- vs. post treatment: Decreased ALFF of the RVM/TCC |
| Baliki et al, 2008 [82] | Chronic low back pain (CBP) and in knee osteoarthritis (OA) | 11 CBP (29-68 years) and 8 OA patients (54-63 years) | Task-based functional MRI | Analgesic treatment (lidocaine patches) | Pre-treatment in CBP patients: Significant brain activity in medial PFC.    Pre-treatment in OA patients: Significant activations in bilateral thalamus, secondary SSC, insula, cingulate cortices, and unilateral activity in the putamen and amygdala. |
| Fernandez et al, 2019 [112] | N/A | 11 healthy individuals | fNIRS | Acupuncture | Pre- vs. post treatment: Subjects exhibited strong activations and distinctive cortical networks associated with SSC. |
| Bandeira et al, 2019 [113] | N/A | 15 healthy male adults (20-55 years) | fNIRS | Peripheral electrical stimulation | Active vs. sham device: Significant activations of right DLPFC and left SMC in the active group but not in the sham group. |
| Gentile et al, 2019 [115] | Fibromyalgia | 15 patients with fibromyalgia (31-53 years); 9 healthy controls (16-48 years) | fNIRS | N/A | Fibromyalgia patients: Decreased motor cortex activation during movement.  Healthy controls: Increased motor cortex activations during movement. |
| Gentile et al, 2019 [114] | Fibromyalgia | 24 patients with fibromyalgia (23-59 years); 24 healthy controls (22-60 years) | fNIRS | N/A | Patients vs. controls: Significantly lower brain activations in motor cortex during movement tasks. |
| Bembich et al. (2015) [118] | N/A | 40 healthy newborns | fNIRS | N/A | Maternal holding was associated with activations in bilateral SSC and posteroinferior right frontal cortex. |
| Erpelding et al. (2016) [125] | Complex regional pain syndrome (CRPS) | 23 patients with CRPS (mean=13.2 years); 21 healthy controls | Structural MRI and resting-state functional MRI | Intensive interdisciplinary psychophysical pain treatment. | Patients vs. controls: Reduced GM in the primary motor cortex, premotor cortex, SMA, midcingulate cortex, orbitofrontal cortex, dlPFC, PCC, precuneus, basal ganglia, thalamus, and hippocampus.    Pre- vs. post-treatment in patients: Increased GM in the dlPFC, thalamus, basal ganglia, amygdala, and hippocampus, and enhanced FC between the dlPFC and the PAG. |
| Becerra et al. (2014) [126] | Complex regional pain syndrome (CRPS) | 26 children and adolescents with P-CRPS (10-18 years); 12 healthy controls | Resting-state functional MRI | Intensive physical and psychological treatment program | CRPS vs. controls before treatment: Differences of brain activations in the fronto-parietal, salience, default mode, central executive, and sensorimotor networks.    Pre- and post-treatment: Reductions in connectivity in salience, central executive, default mode and sensorimotor networks. |
| Simons et al. (2014) [127] | Complex regional pain syndrome (CRPS) | 12 CRPS patients (10-17 years); 12 healthy controls | Resting-state functional MRI | Intensive  physical-biobehavioral pain treatment | CRPS Patients vs. controls: Enhanced rsFC from amygdala to widespread cortical, subcortical, cerebellar regions in CRPS patients compared to controls.  CRPS patients pre- vs. post-treatment: Reduced hyperconnectivity from left amygdala to motor cortex, parietal lobe, and cingulate cortex. |
| Rocca et al. (2014) [128] | Episodic migraine | 12 pediatric patients with episodic migraine (9-17 years) with and without aura; 15 healthy controls (9-18 years) | Morphometric MRI | N/A | Patients vs. controls: GM atrophy of frontal and temporal lobes, increased volume of the right putamen.    Patients with aura vs. patients without aura and controls: Increased volume of left fusiform gyrus. |
| Faria et al. (2015) [129] | Migraine | 14 girls and 14 boys with migraine (10-16 years); 28 healthy controls (10-16 years) | Structural MRI and resting-state functional MRI | N/A | Boy patients vs. girl patients: Reduced GM in the primary SSC, SMA, precuneus, basal ganglia, and amygdala; decreased precuneus rsFC to the thalamus, amygdala, and basal ganglia and decreased amygdala rsFC to the thalamus, anterior midcingulate cortex, and SMA. |
| Goksan et al. (2015) [131] | N/A | 10 healthy infants (38.6-42.7 weeks); 10 healthy adults (23-36 years) | Task-based functional MRI | Noxious stimulation to foot | Adults and infants had significant brain activity in the same 18 brain regions during noxious stimulation.  The amygdala and orbitofrontal cortex were not activated in infants, but were activated in adults. The bilateral auditory cortices, hippocampus, and caudate were activated in infants but were not activated in adults. |
| Gentile et al. (2020) [124] | Fibromyalgia | 38 patients with fibromyalgia (mean=42.18 years); 21 healthy controls (mean=32..62 years) | EEG-fNIRS | N/A | Patients with fibromyalgia vs. healthy controls: The patient group showed reduced tone of cortical motor areas during a fast finger tapping task; presented slow motor performance during rapid movements; showed smaller evoked responses in experimental tasks. |
| Lebel et al. (2008) [140] | Complex regional pain syndrome (CRPS) | 12 pediatric patients with CRPS (aged 9-18) | Task-based functional MRI | Noxious stimulation to CRPS-affected lower extremity | Following active period of evoked pain (brush and cold stimuli) on affected limb: Increased activation of parietal, contralateral and ipsilateral SSC , anterior and middle cingulate, and parts of ACC. Decreased activation in frontal lobe and parietal lobe, middle cingulate, middle temporal lobe, parahippocampus and hippocampus.  During recovery period (no stimuli) on affected limb: decreased activation in frontal, parietal, temporal cortex, parahippocampus and hippocampus. |
| Erpelding et al. (2014) [134] | Complex regional pain syndrome (CRPS) | 12 CRPS patients (aged 10-17); 12 healthy controls | Resting-state functional MRI | N/A | CRPS patients vs. healthy controls: CRPS patients had significantly reduced rsFC of the medial habenula in primary motor cortex, premotor cortex, SMA, anterior midcingulate cortex, and dlPFC compared with healthy controls. |
| Simons et al. (2016) [132] | Complex regional pain syndrome(CRPS) | CRPS patients (mean=13.7 years); healthy controls (mean=14.1 years) | Task-based functional MRI | Fearful face paradigm | CRPS patients vs. healthy controls: CRPS patients had reduced evoked activation in the precentral gyrus, inferior frontal gyrus, supramarginal gyrus, and postcentral gyrus response to fearful faces. |
| Verriotis et al. (2022) [136] | Peripheral neuropathic pain patients | 17 peripheral neuropathic pain patients (aged 11 to 18) | Resting-state functional MRI | N/A | Neuropathic pain patients vs. healthy controls: Pain patients had stronger negative rsFC between right amygdala and right dlPFC, and stronger positive rsFC between right amygdala and left angular gyrus compared with healthy controls. The stronger negative rsFC and stronger positive rsFC results were correlated to lower pain intensity in pain patients. |
| Pettersson et al (2019) [135] | N/A | 28 healthy newborn infants (mean age = 39.9 weeks) | fNIRS | Hip examination vs heart auscultation test | Hip examination vs. heart auscultation test: Newborns had significantly higher oxygenated hemoglobin bilateral somatosensory cortex during hip examination compared to heart auscultation test. Newborns had greater pain scores (from the Premature Infant Pain Profile-Revised measure) during hip examination compared to heart auscultation. |
| Yuan et al. (2022) [139] | N/A | 31 newborn infants  (aged 1-2 days) | fNIRS | Circumcision | Prefrontal activation significantly increased during noxious events (e.g. local injection) and decreased with non-noxious events (e.g. before incision). |
| Karunakaran et al. (2022) [121] | Patients undergoing catheter ablation | 32 pediatric patients (mean age of 15.8 years) | fNIRS | Catheter radiofrequency (RF) ablation with remifentanil vs. placebo | Placebo-controlled group vs. remifentanil group: Greater oxygenated brain activations in inferior and superior medial frontopolar cortices. |
| Ren et al. (2022) [137] | N/A | 60 healthy newborn infants (37-42 weeks) | NIRS | Arterial blood sampling procedure with white noise vs. no white noise | White noise intervention vs. no intervention: White noise group and no white noise group had significantly lower cerebral oxygen saturation during the procedure compared to baseline. Cerebral oxygenation saturation did not significantly differ between two groups. |
| Bembich et al. (2013) [117] | N/A | 30 healthy newborn infants (38-41 weeks) | NIRS | Heel prick procedure with glucose solution vs. breast-  feeding | Glucose solution vs. breast-feeding group: No significant cerebral oxygenation differences during painful procedure.  Breastfeeding group had less intense pain behaviors (from Neonatal Infant Pain Scale) and greater generalized cortical activation during painful procedures. |
| Olsson et al. (2016) [141] | N/A | 10 newborn infants (26-35 weeks) | NIRS | Blood-sampling procedure (venepuncture) with skin-to-skin contact vs. lying in incubator/crib | Skin-to-skin vs. lying in incubator/crib: contact reduces infants’ oxygenated hemoglobin activation during a painful procedure compared to when lying in the incubator/crib. |
| Beken et al. (2014) [138] | N/A | 25 newborn infants with neonatal jaundice (above 37 weeks) | NIRS | Blood-sampling procedure (venepuncture) with glucose solution vs. sterile water | Glucose group had significantly increased cerebral blood volume after the blood sampling procedure compared to baseline. |
| Holmes et al. (2020) [133] | Peripheral neuropathic pain ankle injury | 24 patients; 12 healthy controls (aged 10-24 years) | Structural and functional MRI, DTI |  | Ankle injury cohort vs. healthy controls: In the ankle injury cohort, there was reduced GM in the bilateral SSC compared to healthy controls. Ankle injury showed decreased rsFC between bilateral PAG and frontal pole, between right nucleus accumbens and frontal orbital cortex and insula.  Ankle injury cohort vs. healthy controls: Ankle injury patients showed significantly higher MD in the left IFL, left parietal SLF, right ATR, right parietal SLF, and right temporal SLF. Significantly lower FA in left parietal SLF; significantly higher RD and AD in the left and right parietal SLF. |

MRI: magnetic resonance imaging; DTI: diffusion tensor imaging; fNIRS: functional near-infrared spectroscopy; EEG: Electroencephalography; ACC: anterior cingulate cortex; GM: gray matter; rsFC: resting-state functional connectivity; FA: fractional anisotropy; MD: mean diffusivity; RD: radial diffusivity; AD: axial diffusivity; mPFC: medial prefrontal cortex; RVM: ventromedial medulla; TCC: trigeminocervical complex; dlPFC: dorsolateral PFC; PCC: posterior cingulate cortex; SMC: sensorimotor cortex; SSC: somatosensory cortex; SMA: supplementary motor area; SLF: superior longitudinal fasciculus; PAG: periaqueductal gray; ATR: Anterior Thalamic Radiation; IFL: inferior longitudinal fasciculus; IFG: inferior frontal gyrus; STG: superior temporal gyrus; ALFF: amplitude of low-frequency fluctuations; CH: Cluster headache; TN: Trigeminal neuralgia; CRPS: Complex regional pain syndrome; OA: Osteoarthritis; CMC: carpometacarpal; CBP: Chronic low back pain
